# Nutritional Ketosis in Parkinson’s Disease — a Review of Remaining Questions and Insights

**DOI:** 10.1007/s13311-021-01067-w

**Published:** 2021-07-07

**Authors:** Alexander Choi, Mark Hallett, Debra Ehrlich

**Affiliations:** 1 Human Motor Control Section, Medical Neurology Branch, National Institute of Neurological Disorders and Stroke, Bethesda, USA; 2 Office of the Clinical Director, Parkinson Disease Clinic, National Institute of Neurological Disorders and Stroke, Bethesda, USA

**Keywords:** Parkinson’s disease, Prognostic biomarkers, Nutritional ketosis, Ketogenic diet, Clinical trial design, Outcomes research

## Abstract

Nutritional ketosis has promise for treating Parkinson’s disease. Three previous studies explored the use of a ketogenic diet in cohorts with Parkinson’s disease, and, while not conclusive, the data suggest non-motor symptom benefit. Before the ketogenic diet can be considered as a therapeutic option, it is important to establish with greater certainty that there is a reliable symptomatic benefit: which symptoms or groups of symptoms are impacted (if non-motor symptoms, which ones, and by which mechanism), what timescale is needed to obtain benefit, and how large an effect size can be achieved? To accomplish this, further investigation into the disease mechanisms based on pre-clinical data and hints from the clinical outcomes to date is useful to understand target engagement and gauge which mechanism could lead to a testable hypothesis. We review research pertaining to ketogenic diet, exogenous ketones, fasting, clinical studies, and theoretical review papers regarding therapeutic mechanisms from direct ketone body signaling and indirect metabolic effects. Through discussion of these findings and consideration of whether the ketogenic diet can be regarded as therapeutically useful for adjunctive therapy for Parkinson’s disease, we identify remaining questions for the clinician to consider prior to recommending this diet.

**Supplementary Information:**

The online version contains supplementary material available at 10.1007/s13311-021-01067-w.

## Introduction

Plasma ketone body levels in normal humans fluctuate between 0 and 0.25 mM, increasing to about 1 mM with prolonged exercise or 24h fast and further rising to 5–7 mM in prolonged fasting by 3 days or more, at which point production equilibrates with consumption [1]. Nutritional ketosis (NK) is a target range of ketone bodies in the plasma from 0.5 to 4.0 mM [2], is augmented by exercise [3, 4] and fasting [1], and may be maintained via a diet with careful restriction of combined protein and carbohydrate to 30% of daily energy expenditure [2]. Ketosis is a physiologic state of a newborn [5], has been described as an enduring diet in pre-agricultural societies [6–8], and was used medically by Hippocrates to treat epilepsy, with expanded use in modern times for this purpose after a seminal study by Russell Wilder of the Mayo Clinic [9]. Ketosis has also gained attention for potential therapeutic use in neurodegenerative disease [10], including Alzheimer’s disease (AD), Parkinson’s disease (PD), and amyotrophic lateral sclerosis (ALS), as a way to potentially slow disease progression by providing greater mitochondrial oxidative phosphorylation with decreased free radical generation. Clinical research has shown acutely increased brain network connectivity [11], improved cognitive function [12, 13], and suggested amelioration of symptoms related to the core dopaminergic deficit in PD including bradykinesia and rigidity [14], pain, mood, and urinary frequency [15]. The symptomatic benefit may be twofold: indirect cognitive gains via insulin sensitization and supply of brain adenosine triphosphate (ATP) and other necessary metabolic precursors for synaptogenesis, and with a sufficient ketosis magnitude, direct signaling effects of ketone bodies, which are found to induce gene expression of antioxidant enzymes, reduce apoptosis through a fasting-like metabolic state, and theoretically augment neurotransmitter synthesis. Therefore, a ketogenic diet with restriction of protein and carbohydrates to 30% of daily energy expenditure is a promising candidate for disease modification with or without ketosis augmentation via fasting and exercise.

Questions to consider in interpreting a study with broad metabolic effects such as dietary or exercise intervention include (a) what is the timeframe for estimated effect, (b) which pathway is engaged, and (c) whether the intervention needs to be continuous or can an episodic/iterative process provide satisfactory effects. The hypothesis of benefit from NK, defined as a diet with target serum beta hydroxybutyrate (BOHB) values of 0.5–4.0 mM [16] based on animal models and related biochemistry, often presupposes ketone bodies are more efficient metabolic fuel than glucose, thus providing increased ATP availability to cells with deficient mitochondrial output. Such a hypothesis suggests a mechanism where benefit might be expected within days of ketogenic diet initiation. This can be distinguished from the signaling effects of ketone bodies that would take longer, on the order of weeks. These would include epigenetic modifications for antioxidant and pro-survival signaling as well as indirect metabolic effects such as inhibiting the nuclear factor kappa light chain enhancer of activated B cells (NFKappaB) [17] and nucleotide-binding domain, leucine-rich-containing family, pyrin domain-containing-3 (NLRP3) [18] inflammasome expression. The magnitude of ketosis achieved could be considered, particularly with the hypothesis that ketone bodies per se play a therapeutic role, as well as measuring metabolic effects of insulin sensitivity and antioxidation. Finally, a platform approach might facilitate testing hypotheses and questions of mechanism involved by manipulations in the intervention, e.g., comparing episodic fasting, exercise, and/or exogenous ketones vs. a continuous metabolic effect of standard ketogenic or medium chain triglyceride (MCT) oil-supplemented diet.

## Existing Literature on Nutritional Ketosis as Clinical Therapy in Parkinson’s Disease

To date, three studies formally evaluated nutritional ketosis in PD subjects. Van Itallie et al. [14] reported results of an open-label feasibility study. Seven participants with idiopathic PD were asked to maintain an at-home strict ketogenic diet (4:1 lipid:protein + carbohydrate grams or 90% lipid kilocalories) for 28 days. Five participants completed the study with mostly good adherence to prescribed menus, while one participant dropped out due to inability to prepare the diet and another withdrew from the study for unrelated reasons. The participants who completed the study had varying degrees of ketosis, with most participants in a high therapeutic range of beta-OHB levels (mean 6.6 mM, range 4.8–8.9 mM) and one outlier with a lower range (1.13–1.56 mM). All five completers exhibited improved total Unified Parkinson’s Disease Rating Scale (UPDRS) score and motor subscores. Although the results suggest that adherence to a strict ketogenic diet for 4 weeks is feasible in patients with PD, this study had a number of limitations. The degree to which the results can be attributed to a placebo effect is unclear given the lack of a control group. Additional limitations include the lack of rater blinding, low frequency of quantitative ketosis monitoring, limited study size, and strict 4:1 standard ketogenic diet regimen, the latter because a less extreme ketogenic diet supplemented by medium chain triglycerides or exogenous ketones might improve adherence. Interpretation of the results is limited due to the drop out of 2 of 7 participants and additional 2 of the 5 remaining participants with reported dietary relapse. Future, larger studies achieving a similar ketosis magnitude compared to a control group would help to better interpret these results.

Phillips et al. [15] addressed some of these knowledge gaps by comparing two randomized groups using a comparatively less stringent ketogenic diet vs. a low-fat diet. The diets were prescribed by meal plans, each by kilocalories approximately 18% protein and either 79% lipid/3.7% net carb (KD) or 23% lipid/59% net carb (low-fat diet), the latter also with greater dietary fiber, stratified by estimated daily energy expenditure. The 8-week study involved daily participant ketosis monitoring by AM fasting beta-OHB levels using a validated fingerstick ketone meter. Dietary monitoring relied on participants to check off each meal from the plan outlined for the study period. Investigators reported reduced MDS-UPDRS-I scores (8.96 ± 4.34 vs 11.15 ± 4.15, *P* < 0.001), particularly urinary, pain/sensory disturbance, fatigue, daytime sleepiness, and cognitive impairment subscores. However, this study also had some limitations including infrequent data capture and diet monitoring relying upon self-report, which precludes correlative analysis between ketosis value and clinical rating scale (UPDRS) that was performed only pre- vs. post-intervention. Another possible weakness was the relatively low mean beta OHB level (1.19) in the KD group. It is probable that greater benefit could be seen with a greater degree of ketosis.

Krikorian et al. [13] evaluated another less stringent ketogenic diet in a PD cohort with mild cognitive impairment (PD-MCI) with unlimited protein and < 20 g carb target. They recognized that impaired glucose tolerance/metabolic syndrome is a risk factor for higher comorbidity in PD than in the general population and that the literature supports a ketogenic diet as a therapy specifically for metabolic syndrome [19]. Krikorian et al. similarly used pre- vs. post-intervention data capture of 8 weeks duration, assessing cognition as well as UPDRS.

Dietary manipulation of only carbohydrates may provide a more feasible approach with a lower degree of dietary change required for participants; however, even the less restrictive guidelines were not strictly adhered to. The investigators followed 7 subjects in the low carbohydrate group and 7 in the high carbohydrate group. Dietary monitoring was done by patients filling out 3-day diaries during each study week. Unfortunately, the low carbohydrate group had a mean carbohydrate daily intake of 36 g (above 20 g) and only a low-level ketosis (mean BOHB 0.31 mM by study end). Despite the low ketosis values, Krikorian et al. [13], nonetheless, found improved short-term memory and verbal fluency in the low carbohydrate group compared to the high carbohydrate diet, despite no motor differences on UPDRS-III or finger-tapping speed.

Summarizing the above studies, improvements in non-motor symptoms were found in studies of ketogenic diet in PD. These included subjective benefits in urinary frequency/urgency, pain, and mood, and improved lexical fluency and word recall in a small PD-MCI cohort (Table 1). While motor benefit was noted in the open label Van Itallie et al. study, UPDRS motor scores showed no significant differences when compared to low-fat diets in the Phillips et al. study and high-carbohydrate group in the Krikorian et al. study. Which mechanisms might be responsible for these reported benefits? Partial placebo and rater bias effects cannot be excluded, given the lack of blinding of either participants or study raters. The resolution of outcome assessment at 1–2 months does not differentiate between immediate effects such as from an ATP supply to deficient mitochondria in vulnerable striatal-thalamic-cortical neuronal populations and later effects from epigenetic metabolic factors on cell survival, oxidative stress, neurotransmitter, hormonal, paracrine, or autocrine signaling. The degree of ketosis reported is one variable, from the stricter ketogenic diet in Van Itallie et al.’s study reporting mean BOHB 5–9 mM vs. Phillips et al.’s and Krikorian et al.’s studies 1.2 mM and 0.3 mM, respectively. The metabolic syndrome impairs ketogenesis with higher mean insulin levels [16] such that lower mean ketosis values are expected for many PD patients, perhaps by 20%; this is a consideration if a threshold effect exists for ketone body signaling. There is a caveat of limited outcomes assessment in these studies. UPDRS is more relevant with clinical trials because of the proven reliability [20] and correlation to disease progression [21]. However, clinical meaningfulness and sensitivity are called into question, with various simple quantitative tests, such as functional reach, timed walk, timed block sorting, and timed dotting, outperforming UPDRS-III motor score in predicting disability, gait instability, falls, depression, dementia, and psychosis over mean 2.4 years follow-up [22].

**Table 1:** Improvements in non-motor symptoms found in studies of ketogenic diet in Parkinson’s disease

Study lead author/year published	Ketogenic diet (KD) design	Primary outcome	Results	Comments
Van Itallie, 2005 [14]	Open-label, single-arm 7 participants KD × 4 weeks	Pilot feasibility in the home; UPDRS performed weekly	Decreases in total UPDRS by approximately 20–40%	Strict ketogenic diet goal, 90% lipids by energy, only 5 participants (after 2 dropouts)
Phillips, 2018 [15]	Open-label, randomized 47 participants to KD vs. low-fat diet (LFD) × 8 weeks	Within and between group mean differences in total MDS-UPDRS scores baseline to 8 weeks	Improvements in MDS-UPDRS I scores (KD within-group − 4.58 ± 2.17). Between-group subjective improvements in urinary frequency, pain, daytime sleepiness, cognitive impairment improvements vs. low-fat diet. No between-group differences for MDS-UPDRS II, III, or IV	Mean BOHB 1.19 mM (attained therapeutic target) 2 dropouts in KD group due to perceived side effects (worsening tremor and/or rigidity)
Krikorian, 2019 [13]	Open-label, randomized 18 participants to KD vs. low-fat diet (LFD) × 8 weeks	Proof of concept effects of NK on cognitive performance in a PD-MCI cohort Outcomes included: MDS-UPDRS-III, finger tapping test, Controlled Oral Word Association Test, verbal paired associate learning test, California Verbal Learning Test	Improvements in lexical access and working memory in KD group compared to LFD group; no between-group differences in MDS-UPDRS-III or finger tapping	Exploratory analysis (no primary outcome or power analysis specified) Low mean ketosis in KD group (0.31 mM at week 8) Non-standardized protein, higher intake in low-carb group (123 g vs. 89 g mean daily protein) 3 dropouts in KD group (1 for perceived side effect)

*UPDRS* Unified Parkinson’s Disease Rating Scale, *MDS-UPDRS* Movement Disorder Society Unified Parkinson’s Disease Rating Scale, *BOHB* beta-hydroxybutyrate, *MCI* mild cognitive impairment

## Effects of Nutritional Ketosis on Parkinson’s Disease — Evidence and Theories

One of the key correlates of both aging and insulin resistance is the reduced cerebral metabolic rate of glucose as defined by positron emission tomography (PET) fluorodeoxyglucose tracer [23]. Cerebral metabolic rate can be gauged indirectly via functional magnetic resonance imaging (fMRI) blood oxygen level-dependent (BOLD) signal. One measure, the amplitude of low-frequency (0.01–0.1 Hz) fluctuations (ALFF), is correlated with cerebral blood flow [24] and studied as a possible index of regional network stability over time. As described by reviews on fasting NK [25, 26], there are benefits associated with fasting in rodent models of learning and memory associated with PI3K via cyclic AMP response element binding protein **(**CREB) and brain-derived neurotrophic factor (BDNF) pathways activated in this state as an evolutionary adaptation for homeostasis and survival. Such pathways provide a mechanistic theory for learning and memory involving brain network stabilization, which may be a target for NK therapy. Evidence for a similar benefit was found via insulin administration, and similarly by fasting, ketogenic diet, and exogenous ketone ester drink, as described below.

Glycogen stores provide glycolysis during fasts for ~ 30 h. However, brain glycogen utilization prefers glial stores, and ^13^C magnetic resonance spectroscopy (MRS) showed these become depleted in 5–10 h [27]. Therefore, a typical overnight fast involves relative endogenous brain ketosis even if not detected by peripheral blood ketones. Accordingly, a study examined ketosis effects on fMRI connectivity using Louvain parameter-free modulation maximization to derive network stability, which showed an inverse correlation with age, specifically an S-shaped decrease at ages 50–70, and an inverse correlation with a cognitive test, Mini-Mental Status Exam, in separate population cohorts [11]. Participants were scanned after an overnight fast from a carbohydrate predominant usual diet compared to a group on a ketogenic diet for 1 week; a group receiving a ketone ester drink; and a control group after the usual carbohydrate predominant meal, and found that overnight fast led to statistically equivalent increased global stabilization to ketogenic diet for 1 week. Similarly, the index ALFF correlated with the network stability measure and was increased in the ketogenic diet and overnight fasting groups compared to the usual diet in the resting state as well as motor and spatial tasks over 56 min, specifically related to modular network reorganization rather than change in connection strength of existing nodes. While PET studies show age-related reduction in cerebral metabolic rate of glucose but not in cerebral uptake of oxygen (O_2_) or cerebral blood flow, the cerebral metabolic rate of ketone body acetoacetate is constant [28], while the uptake rate constant (Kacac) is reduced, indicating again separation between brain and peripheral ketone systems and relative preservation of brain ketosis metabolism, as previously found using the arterial-venous difference method [29, 30]. Accordingly, NK appears to be a worthy candidate for clinical research in aging-related neurodegenerative disorders, which are characterized by bioenergetic failure and network instability, as a therapeutic tool to augment network stability and preserve or restore cognitive function.

Evidence indicates PD pathobiology is driven by deficient function, structural localization, and recycling of mitochondria and related superoxide/nitrite radical formation [31]. Pre-clinical data of a ketogenic diet in rats in a traumatic brain injury model showed improved cortical neuronal respiratory capacity, where reduced mitochondrial complexes II–IV related to nitric oxide/superoxide radical production was ameliorated in the ketogenic diet group [32]. Similarly, dextro-beta hydroxybutyrate (d-BOHB) administered to cortical neurons in vitro [33], and subcutaneously infused in mice, lessened dopaminergic neuron cell death induced by neurotoxin 1-methyl-4-phenyl-1,2,3,6-tetrahydropyridine (MPTP) [34]. Ketogenic diets given to mice protected dopaminergic neurons from another neurotoxin, 6-hydroxydopamine (6-OHDA) [35]. Both MPTP and 6-OHDA induce mitochondrial complex I deficiency in mice, and gene expression of complex I is especially deficient in PD substantia nigra at post-mortem [36], associated with increased superoxide radical formation. Another feature of ketosis is increased supply of nicotinamide adenine dinucleotide phosphate (NADPH) by decreased glycolysis and concomitant shuttling of pyruvate via the pentose phosphate pathway (PPP), therefore increasing the supply of reduced glutathione (GSH) available to remove hydrogen peroxide–derived free radicals [10]. Given that NADPH is decreased in the putamen at post-mortem in early PD, as defined by the McKeith Lewy Body score [37], and that oxidative stress is a sequel of complex I inhibition in PD [38], there appears to be a role for NK to slow disease progression. When such PD models are combined with the existing clinical data reviewed above that show improved UPDRS scores, one may surmise there would be a direct effect of ketone bodies to support a deficient but not yet degenerated population of dopaminergic neurons in the substantia nigra and their synaptic partners in the striatum. This would attenuate globus pallidus pars externa (GPe) and subthalamic nucleus (STN) outflow and augment cortical globus pallidus pars interna (GPi) modulation. The premise that such functionally impaired neurons exist in PD is supported by post-mortem analysis described in the seminal paper of Kordower et al. [39], showing nearly complete loss of tyrosine hydroxylase reactivity in the dorsal putamen in patients with 4–5 years disease duration, whereas a small subpopulation of melanin-positive and tyrosine hydroxylase-positive substantia nigra pars compacta neurons usually remained [40]. The theory that NK may slow PD progression is gaining support, since cell death in neurodegenerative disease, including PD, relates to inadequate calcium buffering by dysfunctional mitochondria in specific neuronal populations, leading to mitochondrial permeability pore formation, depolarization, and programmed cell death [41, 42]. Therefore, supporting mitochondria through energy supply is an attractive neurotherapeutic strategy.

During fasting periods or ketogenic diets, the metabolic shift to fatty acid oxidation transiently upregulates mitochondrial free radical formation and subsequently induces gene expression of antioxidants, as well as increases nicotinamide adenine dinucleotide (NAD +) in the cytoplasm, together which are thought to promote organism survival and resilience to free radical stress. Acetyl coenzyme A (acetylCoA) metabolic supply to the brain switches progressively from glycolysis to ketone bodies by mitochondrial 3-hydroxy-3methyl-glytaryl COA synthase (HMGCS2), an enzyme which is upregulated by peroxisome proliferator–activated receptor (PPAR), a fatty acid receptor expressed predominantly in the liver, from the switch to lipid metabolism [43]. Increased fat oxidation is related to reduced triglyceride stores and serum triglyceride levels and increased mitochondrial oxygen consumption with concomitantly increased mitochondrial density and mitochondrial reactive oxygen species (mtROS) and antioxidant activity, superoxide dismutase (SOD), and catalase, as metabolism shifts from glycolysis. In the animal model *Caenorhabditis elegans*, inhibition of glycolysis (modeling the fasting state) leads to increased ROS at day 2 and catalase activity at day 6 with reduced mtROS at 120 h and increased lifespan and survival after administering mitochondrial electron transport chain inhibitors sodium azide and paraquat [44]. The survival benefit was lost by pretreatment with the antioxidant N-acetylcysteine. This supports the adaptive benefit of a basal level of oxidative stress to upregulate antioxidant defense, which is primarily mitochondrial derived from ETC complexes I and III production of superoxide radical (O_2_^•−^), referred to as mitohormesis [45, 46]. A key signaling effect of BOHB is inhibiting histone deacetylase (HDAC) classes I and II associated with increased expression of forkhead box O (FOXO) 3a. The latter induces metallothionein II, SOD2, and catalase [47], with consistent findings of *C. elegans* lifespan extension [48], and protection from paraquat-induced lipid peroxidation and protein carbonyls and nitration (4-HNE) [49]. These signaling effects of ketone bodies support using NK in a clinical trial with the goal to slow PD disease progression.

**Fig. 1:**
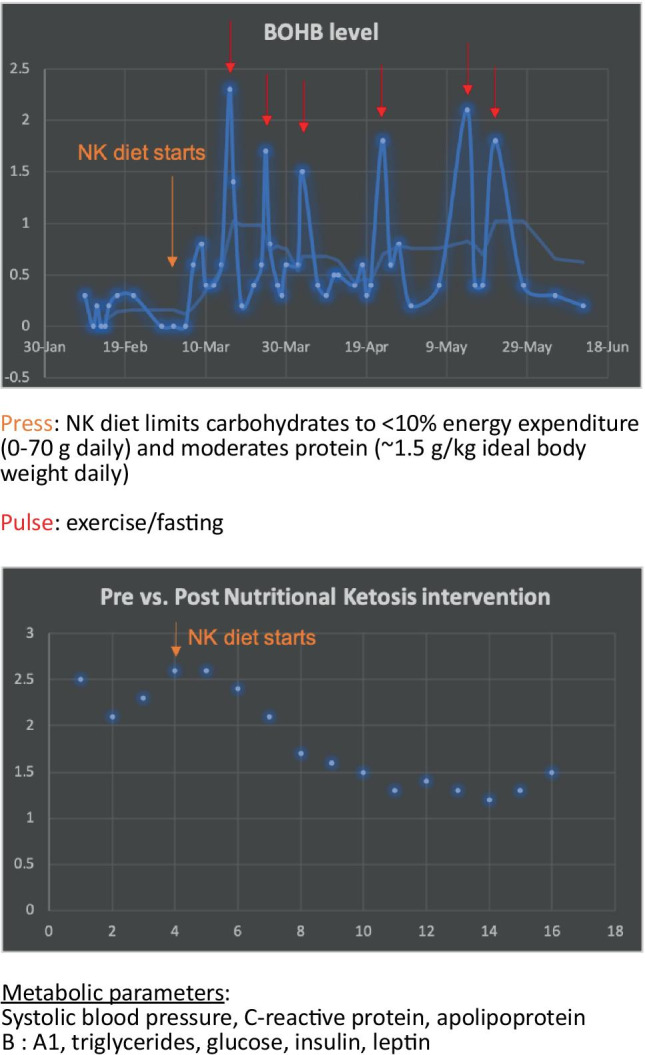
Press and pulse model for neurometabolic degenerative disease. Hypothetical intervention begins at week 4 in the home (orange arrow, “NK diet starts”) with ketone meter, BP cuff and weekly lab testing, showing improved metabolic syndrome parameters. Instructions to fast (time-restricted feeding or intermittent) and/or exercise produces episodically elevated ketosis (red arrows)

While the pre-clinical data are potentially exciting regarding a potential role of NK in slowing neurodegenerative disease, they raise the question of how mechanisms found in mitochondrial toxicity and ketone body models might translate to the reported clinical benefits in the PD NK trials. The mechanisms underlying these reported benefits in open-label studies can be divided into fasting (caloric restriction) vs. ketogenic diet, immediate vs. delayed, and possibly into a threshold effect related to the intensity of the intervention and repeated suprathreshold ketogenic episodes. The latter may characterize the difference between fasting and a ketogenic diet or may be a factor within each intervention. For instance, it is believed that fasting promotes autophagy to a higher degree than a ketogenic diet due to the higher levels of AMPK achieved. Clinically, interspersing the two interventions is proposed via “press + pulse” therapeutic mode to treatment cancer [50], which has been successfully adopted [51]. Of course, fasting differs from a ketogenic diet in terms of reduced energy supply, higher protein catabolism, and a higher degree of ketone body formation relative to fatty acid metabolism as well as hormone signaling, with decreased ghrelin/growth hormone levels and increased leptin hormone levels in the “fed” state. Importantly, intermittent fasting or using a time-restricted feeding window does not imply caloric restriction with the attendant proportionally decreased resting metabolic rate, but rather are pauses in the usual feeding routine where resting energy expenditure is maintained [25]. Exercise also augmented brain ketosis in a study using a cohort with mild AD [52]. Such a press-pulse approach might be adapted to neurometabolic degenerative diseases including PD (Fig. 1). In terms of intensity of intervention, a strict 4:1 ketogenic diet was prescribed in the initial ketogenic diet study in PD by Van Itallie et al. [14], which provided less carbohydrates and protein and increased plasma BOHB to a higher degree than the subsequent studies, possibly relevant to the large effect size reported in the UPDRS motor scores. On the other hand, Mujica-Parodi and colleagues reported that an overnight fast produced a degree of resting state network stability similar to 1 week of a ketogenic diet in healthy volunteers, which they attributed to exhaustion of astrocyte glycogen stores even while 36–48 h of fasting are required for peripheral glycogen total consumption [11]. Answering questions of variability in the data and study design would be best served by well-designed, rater-blinded studies comparing a usual diet to one or more interventions, diet, fasting, exercise, exogenous ketones with adequate washout or in a multi-arm, potentially multi-phase platform trial design. While inconclusive, suggestions of mechanisms underlying specific reported benefits are outlined below.

## The Impact of Nutritional Ketosis on Parkinson’s Disease Functional Domains

Several related domains, including attention, working memory, and task switching appear enhanced by both ketogenic and low carbohydrate diets [13, 53, 54], while increased blood glucose, as per hemoglobin A1c values, is associated with reduced working memory and hippocampal volume and increased mean diffusivity [55]. Therefore, the mechanism is presumably related to the metabolic pathway effected by the switch from carbohydrate to fat as the energy source. One consideration is a pathobiological model described in Alzheimer’s disease (AD) and mild cognitive impairment as “type 3 diabetes” where there is deficient insulin signaling in the brain with downregulation of insulin receptors and insulin-like growth factor 1 (IGF-1), with IGF-1 production in the liver controlled by pulsatile growth hormone release [56]. A pilot study showed cognitive improvement through growth hormone releasing hormone administration in both healthy and MCI participants [57]. Growth hormone signaling is increased by fasting [58] and, therefore, may contribute to cognitive benefits of the metabolic switch involved in fasting and a ketogenic diet. Another relevant factor may be BDNF not only expressed in neurons related to energy homeostasis, such as in the hypothalamus, but also widely expressed throughout the mature brain with release in response to glutamate receptor activation [59].^.^Hippocampal expression of BDNF during exercise in mice and humans is related to improved spatial memory and learning, specifically via cyclic AMP response element binding protein CREB, as evidenced through introduction of a BDNF inhibitor, which blocked both memory/learning and the CREB messenger ribonucleic acid (mRNA) expression effects of exercise [60]. In mice, both intermittent fasting and exercise-associated increased BDNF levels correlated with lower resting heart rate and higher heart rate variability reflecting parasympathetic tone [61, 62]. BDNF levels are proportionately increased in plasma and brain through exercise and independently increased in the same participants in a crossover design using a low-carbohydrate, Paleolithic diet [63]. Accordingly, increased BDNF levels may contribute to memory and learning benefits independently from ketosis.

Further evidence for cognitive benefit from fatty acid metabolism comes from clinical trials in AD using MCT. MCT is metabolized to ketone bodies in the liver by three enzymatic steps with direct intestine to liver portal absorption, found to speed transition to ketosis in an otherwise equal ketogenic diet, and increased levels of blood ketones by around 0.4 mM [64]. A meta-analysis of the use of MCT NK in MCI/AD included 13 trials, of which 7 were randomized with placebo control and 5 measured the same global cognitive score, the Alzheimer’s Disease Assessment Scale-Cognitive Subscale (ADAS-Cog), which, in a pooled Forrest plot, showed statistically significant cognitive improvement [65]. Similarly, insulin administration improved memory performance in early disease stages, mildly cognitive impaired, and mild dementia elderly cohorts, particularly in the apolipoprotein epsilon 4 (APOE4) genotype negative subgroup [66–68]. The memory improvement in these cohorts was followed out to 4 months with attenuated reduction in fluorodeoxyglucose (FDG) PET glucose metabolism [69]. This supports the plasticity of hypometabolic brain because AD involves 20–40% global reduction of glucose uptake by FDG PET imaging, particularly in the hippocampus where glucose utilization is relatively high. Amyloid pathology is clearly implicated in cognitive impairment in PD [70]. Low CSF amyloid beta in a PD cohort correlated independently with incident cognitive impairment and was associated with an earlier age of dementia onset [71]. At post-mortem evaluation, triple deposition of hyperphosphorylated tau, alpha-synuclein, and amyloid beta (Abeta) in the prefrontal cortex and superior/middle temporal lobe gyri was associated with dementia in PD [72]. Further supporting a pathobiological role for Abeta in PD, alpha-synuclein and amyloid beta double transgenic mice showed more Lewy Body pathology than the alpha synuclein gene mutation alone [73]. Abeta PET imaging showed increased cognitive impairment when elevated retention in cortical areas co-occurred with retention in the striatum [74]. Additionally, Pittsburgh B Abeta PET retention was associated with more rapid cognitive decline over 4 years in a PD cohort [75], as was low CSF Abeta42 combined with APOE4 allele over 5 years [76].

Studies showed ADAS-Cog score improvement in the APOE4 negative subgroup only on post-hoc analysis [77, 78]. APOE4 is associated with low-density lipoprotein (LDL) receptors, cerebral amyloid angiopathy, and formation of astrocyte lipid core formation with less transfer of cholesterol to neurons [79], thus suggesting a pathobiological risk that is distinct from brain insulin resistance and glucose hypometabolism. It is known that insulin resistance, as in metabolic syndrome and type 2 diabetes mellitus, is associated with hippocampal atrophy, particularly in the dentate gyrus with atrophic spine arborization. Therefore, promoting insulin sensitization, whether or not strictly ketogenic, may be beneficial with regard to encoding and recall, mood, and other related cognitive processes. Furthermore, a more stable energy source may be beneficial for fatigue, pain and other “non-motor” symptoms.

**Fig. 2:**
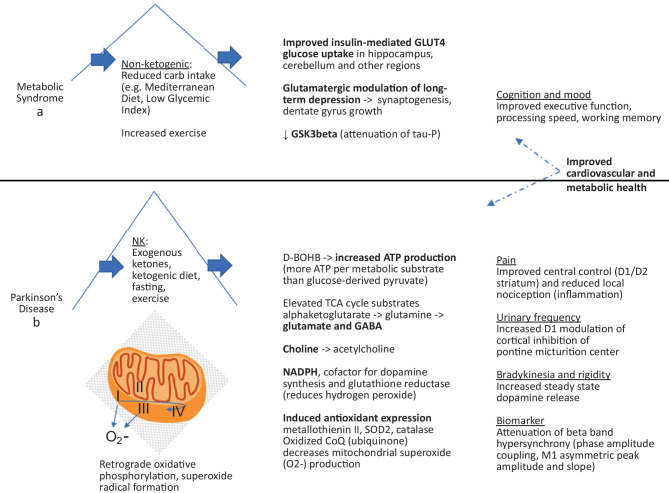
**a** Benefits attributed to reduced carbohydrate intake similar to increased activity levels include improved cognition and mood.** b** Benefits attributed to a ketogenic diet in PD may relate to increased dopamine synthesis and availability

A takeaway from seminal studies of insulin or D-BOHB bath to a working perfused rat heart [80] is that both solutions yielded similar additive efficiency gains. Particularly, both solutions resulted in a 12–18-fold elevation in acetyl CoA substrate. Accordingly, it is not ketogenesis per se and the physiologic ketone body D-BOHB that result in a higher mitochondrial ATP output, but rather ketosis or insulin sensitization. While the brain’s glucose supply is largely via insulin-independent means, insulin is known to mediate glucose entry into the brain via glucose transporters (GLUT) 4 and 8 in specific areas of the cortex, hippocampus, hypothalamus, cerebellum, and olfactory bulb, which appears relevant for glutamatergic modulation of long-term depression (LTD)–related learning and memory. This has a significant implication for dietary strategy, as diets which are beneficial for metabolic syndrome and promote insulin signaling, even while not ketogenic, may confer a significant advantage (Fig. 2a). This is borne out in the scientific literature on cognitive impairment, especially in AD and also in PD motor deficits (e.g., insulin-sensitizing exenatide, an incretin, with positive primary outcome on motor score in phase 2 trial) [81].

Comprising most of the relatively sparse data assessing cognitive performance of metabolic therapy in PD, Krikorian et al. [13] showed that a low carbohydrate diet improved working memory and lexical access, processes related to executive function. The neuropsychological profile of PD commonly involves impaired set-shifting tasks, verbal fluency, and working memory [82]. Therefore, it is plausible that the mechanism of cognitive improvement using KD in PD relates both to striatal signaling to the prefrontal cortex and hippocampal synaptogenesis, which are key features of learning and memory. The finding of improved memory despite lack of improved motor scores with low level of ketosis implies the effects of insulin-sparing and thereby insulin-sensitizing metabolism for learning and memory independent of frontostriatal signaling. Such a dissociation is also suggested by the Phillips et al. study [15], with significant improvement in only non-motor subscales of UPDRS in the ketogenic diet group over a carbohydrate predominant, low-fat diet. The concept that ketosis can be therapeutic for the PD motor symptoms of bradykinesia and rigidity is consistent with the results of the Van Itallie et al. [14] study and mechanistically supported by studies described below.

Reports of improved metabolic parameters — hyperglycemia, insulin resistance, blood pressure, weight, and liver disease — as well as motor, pain, and urinary frequency were reported in studies achieving “therapeutic ketosis.” There are at least a few proposed mechanisms related directly to ketone body signaling (Fig. 2b). One is increased ATP availability combined with increased NADPH precursor for neurotransmitter synthesis [83]. Another is more substrates from the first third of the TCA cycle, including glutamate, a precursor to GABA, and citrate, a precursor to cytosolic acetyl-CoA and acetylcholine synthesis. GABA was employed therapeutically in PD via a glutamic acid decarboxylase (GAD) adeno-associated virus 2 (AAV2) clinical trial [84] and modulates the hyper-oscillatory network of excessive GPe/STN and inadequate cortical GPi modulation. A paired-pulse transcranial magnetic stimulation (TMS) study of healthy volunteers on a ketogenic diet showed increased short intracortical inhibition (SICI), widely attributed to GABA-A signaling, as well as increased central beta power on EEG [85]. GABA agonism may be therapeutic in PD as suggested by a study of zolpidem, a GABA-A agonist, in an early, unilateral untreated PD cohort that showed an increase in primary motor cortex (M1) beta power from the unaffected side and a reduced pathologic beta power from the affected side [86]. The pathologic network involves hyperactive glutamatergic signaling in the STN. A third proposed mechanism is increased production and synaptic sequestration of dopamine due to increased supply of NADPH, a cofactor for tetrahydrobiopterin synthesis, in turn a coenzyme for tyrosine hydroxylase synthesis of levodopa from tyrosine [87]. The related dopaminergic medication and electric stimulation (DBS) responsive symptoms are well established and include bradykinesia and rigidity, pain, and urinary frequency, as detailed below.

Direct effects of dopamine and DBS on nigrostriatal integrity have been simply tested in vivo via biomarkers of the hyper oscillatory “beta band” network shown to correlate with rigidity and bradykinesia scores in PD patients via DBS, dopaminergic medication, and GABAergic medication. These biomarkers include phase-amplitude coupling analysis (e.g., modulation index) and the more recently described waveform analysis (increased “spikiness” of peak amplitude and slope as a summation effect of the oscillation on motor waveform) [88]. A similar analysis is ongoing for a ketogenic diet study in recruitment (NCT04584346).

The central pain matrix as a network of descending cortical modulation has been mapped to the prefrontal cortex, anterior cingulate cortex, insula, amygdala, hypothalamus, periaqueductal gray, rostral ventromedial medulla, and dorsolateral pons/tegmentum [89]. The relation to PD is proposed through dopaminergic modulation involving D2 and D1 receptors in the dorsolateral (sensorimotor) striatum, which when lost produces hyperalgesia to painful stimuli, as in MPTP mice [90]. According with the mouse model, abnormally low pain threshold (hyperalgesia) described in PD is responsive to levodopa medication [91].

Apropos the above, the most common type of pain in PD is musculoskeletal, related to local nociception rather than a centrally-mediated mechanism, which may respond to anti-inflammatory medications such as NSAIDs. A ketogenic diet inhibits the proinflammatory mediator NFKappaB, and is found to reduce inflammation in rats [16]. However, the inverse aspect of a ketogenic diet is that it is low in both carbohydrates and the glycemic index. Higher glycemic loads and insulin resistance are related to chronic, low-grade inflammation. Therefore, diets that are lower in high-glycemic carbohydrates even if not ketogenic, which might be termed “low carbohydrate” or “Mediterranean” diets, may alleviate some pain.

In PD, urinary frequency is commonly related to detrusor hyperreflexia. Pathobiological studies in MPTP mice implicate the basal ganglia in inhibiting the micturition reflex, with amelioration of detrusor hyperreflexia by administering a D1-receptor agonist [92, 93]. A clinical study confirmed improvements of timed dopaminergic medication on urinary frequency rating scale and increased bladder retention on cystometry in PD patients who scored higher on irritative bladder symptoms [94]. Reports of improvement in urinary frequency related to a ketogenic diet may therefore support the hypothesis of improved steady-state dopamine availability and/or reduced pathologic STN and GPe hyperactivity.

Changes in microbiome in PD are increasingly characterized with decreased alpha diversity, a scalar ranging from 0 to infinity referring to the number of species/taxonomic units per sample, thereby quantifying species richness; increased lipid polysaccharide (LPS); decreased production of short chain fatty acids (SCFA); and activation of the inflammasome [95–97] as reviewed [98]. SCFA are key components of maintaining integrity of the gut epithelial barrier and preventing bacterial overgrowth from occurring. In a crossover study of mildly cognitively impaired (MCI) and cognitively normal subjective memory complaint participants (CN) on 6 weeks each of a modified, ketogenic Mediterranean diet (MMD) compared to low fat diet (American Heart Association), MMD appears to be therapeutic with respect to SCFA with increased butyrate stool content, one of the main SCFAs, and changes to microbiome composition in the MCI group, although no changes to alpha diversity of either diet in either group were found [99]. Studies of the microbiome as a surrogate marker of low-inflammatory, therapeutic diets in PD are required to further understand the optimal design and mechanism in future studies.

Considering the deleterious effects of diet, long-term fatty food intake has been linked to dietary fat-induced dysbiosis with decreased microbiota species diversity, increased gram-negative bacteria and LPS, inflammation, epithelial cell impaired metabolism, increased gut permeability, and oxidative stress in studies of obese patients [100]. NK research appears to require further investigation with consideration to specific nutrient profiles and microbiological studies such as analysis of 16S rRNA to assay biodiversity. Such considerations might include what are improved colonic integrity/health such as fermented products, prebiotics/probiotics, intake of vegetable proteins, and deleterious or dysbiotic effects of sweeteners (including artificial ones) and processed foods [101].

## Summary

Studies of a ketogenic diet in neurometabolic degenerative disorders including AD and PD consistently demonstrated improved learning and memory, supported by pre-clinical data showing BDNF and glutamate-mediated hippocampal synaptic integrity and network stabilizing factors related to both ketone body and insulin signaling in the brain. Confirmation of preliminary supportive evidence for benefits on mood, mobility, and specifically in PD of executive and processing speed, pain, tremor, rigidity, and urinary frequency related to striatal networks involving thalamocortical loops requires further studies designed with reliable, quantitative measures above and beyond UPDRS. Of particular relevance are questions regarding timescale; effect size; relation to gut microbiome diversity/attenuation of LPS production; variations of press and pulse approaches between diet, fasting, and exercise; and correlation to magnitude of ketosis as opposed to other parameters of metabolic intervention such as reduced hyperglycemia/insulin resistance-related oxidative stress and microvascular ischemia. Readout from readily available metabolic parameters such as the blood glucose:BOHB and triglycerides:HDL indices may be useful to correlate to indices of frontostriatal dysfunction such as beta power hypersynchrony. Comparison may benefit from a platform trial design approach with adequate washout.

To progress further in the study of ketogenic diet PD, the following questions should be considered:

1. Is there a symptomatic benefit? If so, on what timescale, and how large? A double-blind, placebo-controlled trial could be helpful in exploring these questions (of note, the authors of this paper are currently conducting a clinical trial with secondary and exploratory outcomes aimed to explore symptomatic benefits, NCT04584346). Dietary assessment by 24h recall was reliable and lowers burden on participants. Study of outcomes meaningful to patients including mobility and cognition within the same cohort might better determine whether clinically meaningful symptomatic improvements are seen than clinician rating scales. The frequency of data capture can be augmented by wearable or smart phone app sensors and at-home assessments, which may be a mechanism that can be used to better establish the timescale of symptomatic improvements.
2. Which symptoms respond, if any? Are the magnitudes of improvement similar? Based on the data above, there is a suggestion that non-motor (or perhaps attention, short-term recall, autonomic, pain, and mood) symptoms show improvements. Quantitative testing of the magnitude of improvement in non-motor symptoms would be useful to inform future trials, given greater reliability in smaller cohorts compared to phase 3 trial outcomes, particularly UPDRS.
3. Is there a disease modifying effect? In order to explore potential disease modifying effects, a long-term outpatient study is needed which employs the use of good disease biomarkers. First, the mechanism of action of disease modifying benefit, such as rate of oxygen free radical production, must be defined in terms of PD pathobiology. Plasma BDNF is one potential surrogate marker, as are markers of GABA signaling/efferents, such as by EEG beta power or waveform morphology analysis from the affected motor cortex, MRS, or TMS paired-pulse stimulation SICI, or inflammation/oxidative stress markers 8-hydroxy-2-deoxyguanosine (8-OHdG), advanced oxidation protein products (AOPP), c-reactive protein (CRP), and apolipoprotein B:A1 ratio.

## Supplementary Information

Below is the link to the electronic supplementary material.

Supplementary file1 (PDF 509 KB)

Supplementary file2 (PDF 508 KB)

Supplementary file3 (PDF 508 KB)
